# The Evaluation of Tau Deposition with [^18^F]PI-2620 by Using a Semiquantitative Method in Cognitively Normal Subjects and Patients with Mild Cognitive Impairment and Alzheimer's Disease

**DOI:** 10.1155/2021/6640054

**Published:** 2021-07-09

**Authors:** Attapon Jantarato, Sira Vachatimanont, Natphimol Boonkawin, Sukanya Yaset, Anchisa Kunawudhi, Chetsadaporn Promteangtrong, Jintana Assanasen, Nithi Mahanonda, Chanisa Chotipanich

**Affiliations:** ^1^National Cyclotron and PET Centre, Chulabhorn Hospital, Chulabhorn Royal Academy, Thailand; ^2^Chulabhorn Hospital, Chulabhorn Royal Academy, Thailand

## Abstract

**Background:**

Some studies have reported the effectiveness of [^18^F]PI-2620 as an effective tau-binding radiotracer; however, few reports have applied semiquantitative analysis to the tracer. Therefore, this study's aim was to perform a semiquantitative analysis of [^18^F]PI-2620 in individuals with normal cognition and patients with mild cognitive impairment (MCI) and Alzheimer's disease (AD).

**Methods:**

Twenty-six cognitively normal (CN) subjects, 7 patients with AD, and 36 patients with MCI were enrolled. A dynamic positron emission tomography (PET) scan was performed 30–75 min postinjection. PET and T1-weighted magnetic resonance imaging scans were coregistered. The standardized uptake value ratio (SUVr) was used for semiquantitative analysis. The P-Mod software was applied to create volumes of interest. The ANOVA and post hoc Tukey HSD were used for statistical analysis.

**Results:**

In the AD group, the occipital lobe had a significantly higher mean SUVr (1.46 ± 0.57) than in the CN and MCI groups. Compared with the CN group, the AD group showed significantly higher mean SUVr in the fusiform gyrus (1.06 ± 0.09 vs. 1.49 ± 0.86), inferior temporal (1.07 ± 0.07 vs. 1.46 ± 0.08), parietal lobe, lingual gyrus, and precuneus regions. Similarly, the AD group demonstrated a higher mean SUVr than the MCI group in the precuneus, lingual, inferior temporal, fusiform, supramarginal, orbitofrontal, and superior temporal regions. The remaining observed regions, including the striatum, basal ganglia, thalamus, and white matter, showed a low SUVr across all groups with no statistically significant differences.

**Conclusion:**

A significantly higher mean SUVr of [^18^F]PI-2620 was observed in the AD group; a significant area of the brain in the AD group demonstrated tau protein deposit in concordance with Braak Stages III–V, providing useful information to differentiate AD from CN and MCI. Moreover, the low SUVr in the deep striatum and thalamus could be useful for excluding primary tauopathies.

## 1. Introduction

The pathogenesis of neurodegenerative diseases is still being investigated. Substances being considered as hallmarks of neurodegenerative diseases include beta-amyloid (A*β*) plaques and neurofibrillary tangles (NFTs) generated from hyperphosphorylated tau proteins [1]. *In vivo* imaging for detecting tau protein accumulation using positron emission tomography (PET) has shown high potential for early diagnosis [2]. Accumulated NFTs could be visualized even in the early phases of the disease. Furthermore, the clinical presentation and tau protein deposition phase have been reported to coincide [3].

In the past decade, several PET radiopharmaceuticals have been developed. For example, [^18^F]THK5351 or THK5371, [^18^F]AV-1451 (Flortaucipir), and [^11^C]PBB3 have been examined for their biophysical properties and clinical use [4–6]. There have been numerous reports on these radiotracers being highly selective tau proteins. However, these “first generation” tau radiotracers also identify “off-target” accumulations as well [7]. A recently developed “second-generation” tau tracer, [^18^F]PI-2620, binds to all types of tau deposits (3R, 4R, and 3R/4R). Moreover, the tracer has represented outstanding clinical potential for differential diagnosis among A*β*+AD and A*β*+non-AD tauopathies, cortical basal syndrome (CBD), and progressive supranuclear palsy (PSP) [8–12]. However, it has a lower rate of off-target binding and may improve our capabilities for tau detection [13–15]. Consequently, the radiotracer has been considered orphan drug designation diagnosis of PSP and CBD from the European Commission and two by the U.S. Food and Drug Administration.

Although the preclinical and clinical effectiveness of [^18^F]PI-2620 has been studied and demonstrated for a while, only a few studies have focused on the semiquantitative assessment of tau protein in relation to the clinical state of subjects [13]. Consequently, this study is aimed at performing a semiquantitative analysis of [^18^F]PI-2620 in cognitively normal subjects and those with mild cognitive impairment (MCI) and AD.

## 2. Materials and Methods

This study was approved by the Human Research Ethics Committee of Chulabhorn Research Institute. Written informed consent was obtained from all participants before the study.

### 2.1. Participants

A total of 69 subjects were enrolled in the study, with 26 CN subjects, 7 patients with AD, and 36 with MCI. There were 8 men and 18 women in the CN group, aged between 56 and 71 (mean ± SD: 63.81 ± 4.6) years. The AD group consisted of 3 men and 4 women aged from 59 to 74 years (mean ± SD: 64.57 ± 6.09). The MCI group comprised 11 men and 25 women aged 56–85 years (mean ± SD: 66.61 ± 5.92). The individuals in the CN group were verified by neurologists and neuropsychiatrists. They had no history of psychological or neurological diseases, psychotropic drug use, or cancer within the last 5 years. They also scored 24–30 on the Thai version of the Montreal Cognitive Assessment. Subjects with AD were assessed and diagnosed by clinicians, using the National Institute on Aging-Alzheimer's Association criteria for probable AD. Magnetic resonance imaging (MRI) was performed for all participants.

### 2.2. Procedure

All participants underwent tau PET with [^18^F]PI-2620 using a Siemens PET/CT Biograph Vision scanner in a 3D mode.

### 2.3. [^18^F]PI-2620 Imaging Procedure

Dynamic imaging was performed 30 min after an intravenous injection of 185 MBq (5 mCi) of [^18^F]PI-2620. Dynamic brain PET/CT images were obtained for 45 min, and brain CT images were acquired for attenuation correction. Image acquisition parameters included matrix size = 440, zoom = 2, and an all-pass filter. Image reconstruction was performed in 9 frames, at 5 min per frame, using the True X (point spread function reconstruction) plus time-of-flight reconstruction with 8 iterations and 5 subsets. All iterative reconstruction images were used for semiquantitative analysis.

### 2.4. MRI Acquisition

T1-weighted MRI (T1MRI) data were acquired for all participants using an Ingenia 3.0-T Philips MRI system. The parameters for the 3D T1MRI included voxel size of 0.43/0.43/1.20 mm; no overlapping; TR of 6.4 ms; and TE of 3.0 ms, which reconstructed to 512 × 512 over a field of view of 220 × 200 mm.

### 2.5. Data and Statistical Analysis

Data processing and analysis of the PET images were conducted using the P-Mod Neuro tool (PMOD Technologies, Switzerland). [^18^F]PI-2620 PET images were automatically coregistered for each individual using the automatic voxel of interest (VOI) method. The PET images were then registered to the T1MRI data from each subject. The T1MRI data were used for the registration and delineation of the brain reference regions; the data were standardized to the Montreal Neurological Institute (MNI) T1MRI template atlas. VOIs were automatically outlined on the normalized MRI based on the maximum probability following the automated anatomical labeling-merged atlas. The standardized uptake value ratios (SUVr) of [^18^F]PI-2620 were analyzed for various brain regions using the cerebellum as a reference region. Eighteen regions were assessed, including the hippocampus; fusiform gyrus; middle temporal, inferior temporal, superior temporal, supramarginal, orbitofrontal, posterior cingulate, parahippocampus, lingual, occipital, precuneus, parietal, and caudate regions; putamen; thalamus; basal ganglia; and white matter.

Microsoft office 365 and Stata 12 (Stata Corp, USA) were used for all analyses. ANOVA and then pairwise comparisons using post hoc Tukey-HSD were performed to assess tracer uptake in various cortical regions in all groups. A *p* value of <0.05 was considered to indicate statistical significance. We created box and whisker plots for the SUVrs of each region in the 3 groups.

## 3. Results

**Figure 1:**
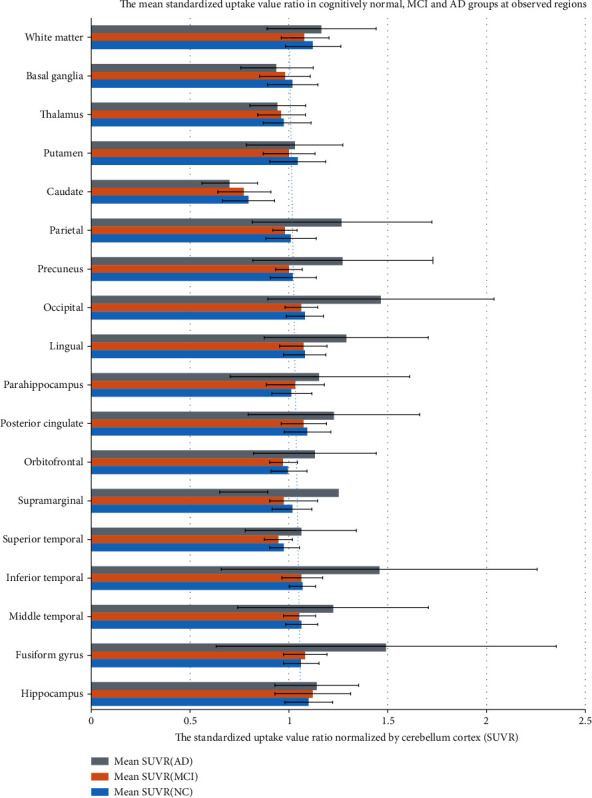
Comparison of the mean SUVr ± SD of various brain regions among the CN, MCI, and AD groups.

**Figure 2:**
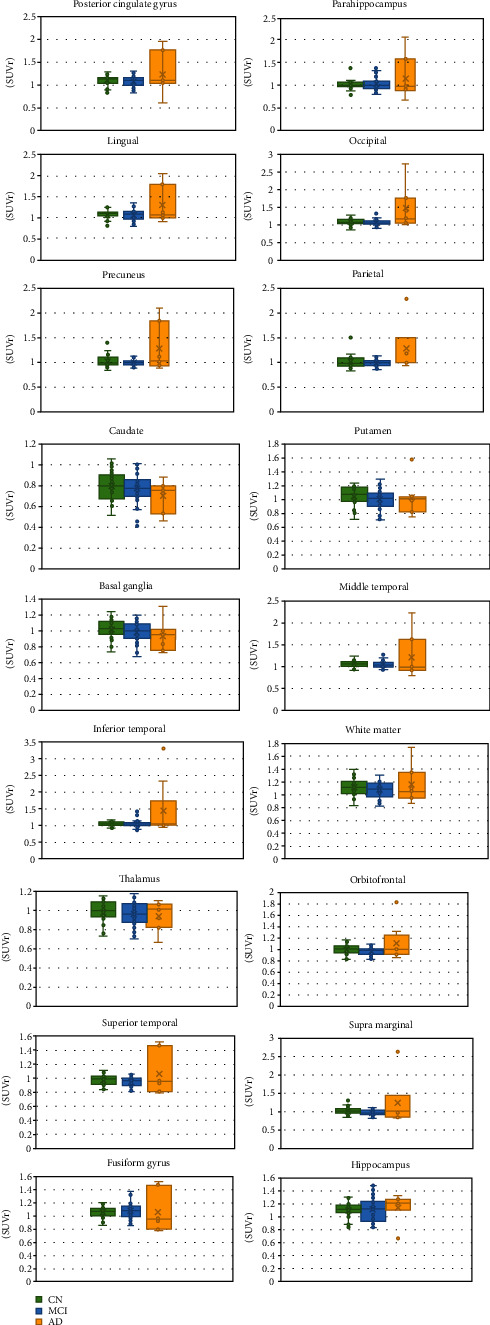
Box and whisker plots comparing SUVr among the CN, MCI, and AD groups in observed regions of the brain.

The demographic characteristics of the study subjects are summarized in Table 1. In addition, compared SUVr plus standard deviation as well as box and whisker plot for the NC, MCI, and AD groups at observed regions are shown in Figures 1 and 2.

The mean SUVr with standard deviation along with the *p* value/*f* value acquired from ANOVA and the *p* value analyzed from pairwise comparison Tukey-HSD test of all groups are shown in Tables 2 and 3.

### 3.1. CN vs. MCI

**Figure 3:**
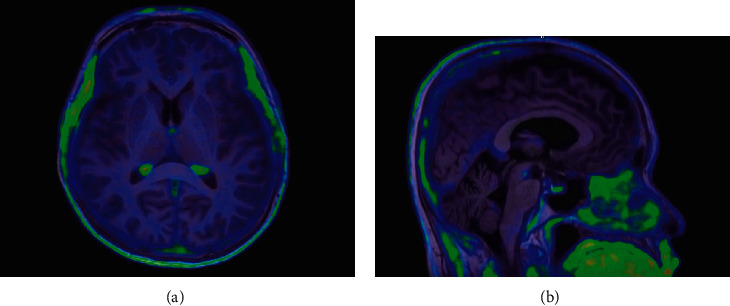
Fusion images between [^18^F]PI-2620 PET and T1-weighted MRI in a CN subject. (a) No uptake of [^18^F]PI-2620 in the bilateral striatum, thalamus, white matter, and whole cortical region. (b) Subtle off-target binding in the midbrain and cingulate gyrus.

Regarding to pairwise comparison between SUVr of NC and MCI, there were none of brain regions showing statistically significant difference. Comparably, the SUVr ranges for all regions of NC and MCI are 0.8-1.2 and 0.77-1.12, respectively. The identical lowest deposition for both groups was observed at caudate. The highest uptake region was marked differently, for the NC was at the white matter and for MCI the SUVr was at the hippocampus. Additionally, the NC group visual analysis demonstrated no uptake at the bilateral striatum, thalamus, white matter, and whole cortical region as well as subtle off-target binding in the midbrain and cingulate gyrus, as displayed in Figure 3.

### 3.2. CN vs. AD

**Figure 4:**
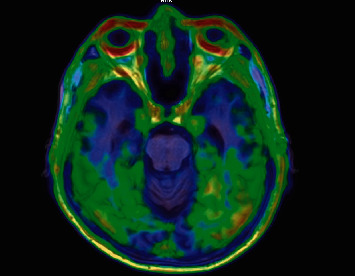
Fusion images between [^18^F]PI-2620 PET and T1-weighted MRI in an AD patient. Intense tau deposition was observed in the bilateral temporal and occipital regions.

The CN and AD groups demonstrated statistically significant SUVr differences in the lingual, precuneus, occipital, parietal, inferior temporal, and fusiform gyrus regions with pairwise *p* values of 0.016, 0.003, 0.02, 0.019, 0.024, and 0.024, respectively. The highest uptake of AD group was demonstrated at the fusiform gyrus while the strongly significantly different SUVr is at the occipital lobe which consists of the visual analysis reveling the intense uptake shown in Figure 4. Besides, the remaining areas are shown in Table 2 and were not statistically significantly different.

### 3.3. MCI vs. AD

The pairwise comparison outcomes between these 2 groups were consistent with NC vs. AD pairwise outcomes. The statistically significant differences were still noted at the fusiform gyrus, inferior parietal, lingual, occipital, precuneus, and parietal regions with *p* values of 0.011, 0.009, 0.007, 0.000, 0.001, and 0.019, respectively. On top of that, there were 3 more regions presenting a significant difference including the superior temporal, supra marginal, and orbitofrontal regions with *p* value of 0.045, 0.012, and 0.013, respectively. Otherwise, the remaining areas SUVr and *p* value were not discovered statistically significantly different as shown in Table 2.

## 4. Discussion

Our study results showed that brain regions significantly linked to the early deposition of phosphorylated tau protein in AD pathogenesis were demonstrated to have significantly higher SUVrs in the AD group than in the MCI and normal groups. These were the occipital, fusiform gyrus, inferior parietal, lingual, occipital, precuneus, and parietal regions (*p* < 0.05). Various neurodegenerative diseases are associated with the intracellular deposition of phosphorylated tau proteins in the brain tissue. The tau aggregation is found in different isoforms, such as 4R or 3R or mixed 3R and 4R [16], predominantly seen under the microscope in AD patients in cross-sectional brain tissue studies. [^18^F]PI-2620 has outstanding binding capacity to both 3R and 4R aggregated tau isoforms. This has been associated with previous studies focusing on the distribution of [^18^F]PI-2620 in AD patients. For instance the report from Mueller et al. [14], who similarly reported higher SUVr at 45–75 p.i. at the fusiform gyrus, inferior temporal, and occipital regions with 1.63 ± 0.29, 1.80 ± 0.40, and 1.45 ± 0.23. Likewise, Barret et al. [17] demonstrated higher SUVrs in the temporal, parietal, precuneus, and cingulate regions in the AD group with an SUVr range of approximately 2.5–2.8, whereas Stephen et al. [18] reported extremely high SUVrs, up to 4, in the abnormal regions. Villemagne et al. [19] and Mormino et al. [20] also described a higher SUVr in the temporoparietal and posterior cingulate regions of subjects with AD.

Although most studies report significantly higher SUVr for patients with AD, a noticeable difference between SUVr and other examined regions still be observed across all publications. The different SUVr values could be linked to variations in the study design that are technical (e.g., imaging parameters for semiquantification analysis, variation in brain-template, or delineation for identification of brain regions of interest) or biological (e.g., the ethnic and brain volume) [21–23]. Therefore, the direct comparison of SUVrs between populations and studies might not be accurate and will need to be standardized for minimization of these variations.

Furthermore, our results showing higher SUVrs in the fusiform gyrus, inferior temporal, lingual, occipital, precuneus, and parietal regions were also consistent with the Braak Stage III–V description of deposition from the temporal portion of the fusiform gyrus (stage III) to the inferior temporal region (stage IV) and then extending to the direction of the occipital region (Stage V) [23, 24]. Considering that regional tau deposition in AD correlates with clinical symptoms and this abnormal pattern of tau deposition correlates with the Braak Stages, [^18^F]PI-2620 can be assumed to be clinically useful for the detection of tau deposits in specific brain regions.

Apart from discovering higher tau deposition in the AD group, our findings also indicated a remarkably low and nonsignificant uptake of [^18^F]PI-2620 in the white matter across all groups. This low uptake emphasized that the tracer was outstanding for both visualization and semiquantification. Tau imaging is still challenging owing to the limitation of off-target binding as seen with the first generation radiotracers such as [^11^C]PBB3, [^18^F]THK-5117, [^18^F]THK-5351, and [^18^F]AV-1451 [25]. The nonspecific retention in the subcortical white matter could be visualized from the utilization of these tracers, resulting in decreased detectability of the early deposition of phosphorylated tau protein in early AD. Second-generation tau imaging tracers such as [^18^F]PI-2620 offer superior performance owing to their higher specificity and sensitivity.

Our study also identified the lowest mean SUVr in the caudate lobe. It was also distinctively low in the deep gray matter structures the thalamus and putamen, with no significant differences among the groups. This finding was consistent with the report by Kroth et al. [13], who described [^18^F]PI-2620's tau-binding properties in a preclinical *in vivo* study, with no off-target binding involving A*β* or MAO-A and MAO-B being the enzyme generally found in the basal ganglia, thalamus, or midbrain. The study, which used semiquantification correlated with visualization, emphasized that the selective binding capacity of [^18^F]PI-2620 could detect primary tauopathy diseases such as progressive supranuclear palsy (PSP) [8], which is associated with significant tau deposition in the globus pallidus, midbrain, and basal ganglia [26].

The comparable SUVr values between CN and MCI subjects in most of the observed regions are notable. Previous studies have determined that the degree of tau deposition is an index of AD severity. It reflects a higher affinity for the tau tracer, correlating with the loss of brain function [27]. Conversely, the mean SUVr in all observed regions of the brain in the CN and MCI groups in this study was not statistically significantly different. This disagreement with previous study results may be owing to various factors [28], for example, the diversity of the observed population resulting to variation of brain structure or slightly loss of brain volume. In addition, a technical factor involving the semiquantification software smoothing of the scans of individuals with MCI with subtle focal uptake results in a lower SUVr.

Similarly, the lack of difference in SUVr between the AD and CN groups for regions including the superior temporal, supramarginal, and orbitofrontal areas, which were significantly higher than that in the MCI group, might be ascribed to the statistical process and imaging semiquantification. Statistically, the pairwise comparison by post hoc Tukey HSD between CN and AD might lead to nonsignificance owing to the AD group's high standard deviation because of the small number of individuals compared with that in the CN group. Notably, the lower SUVr in some cases in the AD group may be due to the severe morphologic changes in the brain structure affecting the brain volume in the small regions analyzed by the P-Mod software. Hence, semiquantification using the imaging tool might be inaccurate, particularly in the voxel analysis of small volumes, leading to individual variations, and should be seriously considered when comparing results with expert visual analysis.

Finally, the semiquantification of [^18^F]PI-2620 by SUVr was beneficial for identifying abnormal tau protein deposition in significant brain regions in patients with AD. In addition, the lower SUVr in the deep gray matter structures may suggest use of the tracer as a tool for primary tauopathies such as PSP. However, this study needs to be repeated on a large scale with patients who are examined for MCI symptoms. Particularly, the longitudinal study for the prodromal AD patients (MCI with low tau) with [^18^F]PI-2620 still needs to be appropriately performed.

## 5. Conclusion

The higher mean SUVr of [^18^F]PI-2620 identified in the AD group in significant brain areas for the diagnosis of AD showed tau protein deposits consistent with Braak Stage III–V classification. In addition, the low background uptake in the white matter provided useful information for the differentiation of AD from CN and MCI. Moreover, the low SUVr in the deep striatum and thalamus could be useful for the exclusion of primary tauopathies such as PSP. The semiquantification of this tracer could be a significant means for improving the sensitivity and specificity of diagnosis of tau-related diseases.

## Figures and Tables

**Table 1:** Participants' characteristics.

	Cognitively normal individuals	Mild cognitive impairment patients	Alzheimer's disease patients
Number	26	36	7
Age: mean ± SD (range), years	63.81 ± 4.6 (56-71)	66.61 ± 5.92 (56-85)	64.57 ± 6.09 (59-74)
Gender (%)			
Men: number (%)	8 (30%)	11 (30%)	3 (43%)
Women: number (%)	18 (70%)	25 (70%)	4 (57%)
Education years: mean (range)	13.4 (0-21)	10.7 (0-18)	11.0 (4-16)
MMSE: mean (range)	27.3 (26-30)	26.1 (20-30)	18.4 (12-23)

**Table 2:** Mean SUVr with SD as well as *p* value and *f* value from one-way ANOVA test in cognitively normal (CN), mild cognitive impairment (MCI), and Alzheimer disease (AD) groups.

Regions	NC		MCI		AD		*p* value	*F* ratio
	Mean SUVR	SD	Mean SUVR	SD	Mean SUVR	SD		
Hippocampus	1.1	0.12	1.12	0.19	1.14	0.21	0.83	0.19
Fusiform gyrus	1.06	0.09	1.08	0.11	1.49	0.86	≤0.001	6.09
Middle temporal	1.06	0.08	1.05	0.08	1.22	0.48	0.07	2.80
Inferior temporal	1.07	0.07	1.06	0.10	1.46	0.80	≤0.001	6.38
Superior temporal	0.98	0.08	0.95	0.07	1.06	0.28	0.06	2.99
Supramarginal	1.02	0.10	0.97	0.07	1.25	0.60	0.01	5.16
Orbitofrontal	1.00	0.09	0.97	0.07	1.13	0.31	0.02	4.37
Posterior cingulate	1.10	0.12	1.07	0.11	1.23	0.43	0.13	2.07
Parahippocampus	1.01	0.10	1.03	0.15	1.16	0.45	0.52	1.44
Lingual	1.08	0.11	1.07	0.12	1.29	0.41	0.01	4.91
Occipital	1.08	0.09	1.06	0.08	1.46	0.57	≤0.001	11.75
Precuneus	1.02	0.12	1.00	0.07	1.27	0.45	≤0.001	7.56
Parietal	1.01	0.13	0.98	0.06	1.27	0.45	≤0.001	8.25
Caudate	0.80	0.13	0.77	0.13	0.70	0.14	0.26	1.39
Putamen	1.04	0.14	1.00	0.13	1.03	0.24	0.53	0.65
Thalamus	0.98	0.11	0.96	0.12	0.94	0.14	0.66	0.41
Basal ganglia	1.02	0.13	0.98	0.13	0.94	0.18	0.31	1.19
White matter	1.12	0.14	1.08	0.12	1.16	0.28	0.33	1.13

**Table 3:** *p* value from pairwise comparisons using post hoc Tukey HSD test among the cognitively normal (CN), mild cognitive impairment (MCI), and Alzheimer disease (AD) groups.

Regions	NC vs. MCI	MCI vs. AD	NC vs. AD
	*p* value	*p* value	*p* value
Hippocampus	0.714	0.745	0.503
Fusiform gyrus	0.551	0.011	0.024
Middle temporal	0.563	0.060	0.133
Inferior temporal	0.898	0.009	0.024
Superior temporal	0.133	0.045	0.189
Supramarginal	0.055	0.012	0.073
Orbitofrontal	0.126	0.013	0.094
Posterior cingulate	0.500	0.086	0.194
Parahippocampus	0.610	0.198	0.162
Lingual	0.399	0.007	0.016
Occipital	0.485	≤0.001	0.003
Precuneus	0.348	0.002	0.020
Parietal	0.212	0.001	0.019
Caudate	0.473	0.220	0.110
Putamen	0.212	0.641	0.853
Thalamus	0.457	0.756	0.456
Basal ganglia	0.259	0.484	0.207
White matter	0.232	0.214	0.582

## Data Availability

The data that support the findings of this study are available from the corresponding author upon reasonable request.

## References

[1] Serrano-Pozo A., Frosch M. P., Masliah E., Hyman B. T. (2011). Neuropathological alterations in Alzheimer disease. *Cold Spring Harbor Perspectives in Medicine*.

[2] Berti V., Pupi A., Mosconi L. (2011). PET/CT in diagnosis of dementia. *Annals of the New York Academy of Sciences*.

[3] Ossenkoppele R., Schonhaut D. R., Schöll M. (2016). Tau PET patterns mirror clinical and neuroanatomical variability in Alzheimer’s disease. *Brain*.

[4] Lois C., Gonzalez I., Johnson K. A., Price J. C. (2019). PET imaging of tau protein targets: a methodology perspective. *Brain Imaging and Behavior*.

[5] Harada R., Okamura N., Furumoto S. (2016). Characteristics of tau and its ligands in PET imaging. *Biomolecules*.

[6] Okamura N., Harada R., Furukawa K. (2016). Advances in the development of tau PET radiotracers and their clinical applications. *Ageing Research Reviews*.

[7] Leuzy A., Chiotis K., Lemoine L. (2019). Tau PET imaging in neurodegenerative tauopathies--still a challenge. *Molecular Psychiatry*.

[8] Brendel M., Barthel H., van Eimeren T. (2020). Assessment of 18F-PI-2620 as a biomarker in progressive supranuclear palsy. *JAMA Neurology*.

[9] Palleis C., Brendel M., Finze A. (2021). Cortical [^18^F]PI-2620 binding differentiates corticobasal syndrome subtypes. *Movement Disorders*.

[10] Oh M., Oh S. J., Lee S. J. (2020). Clinical evaluation of ^18^F-PI-2620 as a potent PET radiotracer imaging tau protein in Alzheimer disease and other neurodegenerative diseases compared with ^18^F-THK-5351. *Clinical Nuclear Medicine*.

[11] Song M., Beyer L., Kaiser L. (2021). Binding characteristics of [^18^F]PI-2620 distinguish the clinically predicted tau isoform in different tauopathies by PET. *Journal of Cerebral Blood Flow & Metabolism*.

[12] Beyer L., Brendel M. (2021). Imaging of tau pathology in neurodegenerative diseases: an update. *Seminars in Nuclear Medicine*.

[13] Kroth H., Oden F., Molette J. (2019). Discovery and preclinical characterization of [^18^F]PI-2620, a next-generation tau PET tracer for the assessment of tau pathology in Alzheimer’s disease and other tauopathies. *European Journal of Nuclear Medicine and Molecular Imaging*.

[14] Mueller A., Bullich S., Barret O. (2020). Tau PET imaging with ^18^F-PI-2620 in patients with Alzheimer disease and healthy controls: a first-in-humans study. *Journal of Nuclear Medicine*.

[15] Chotipanich C., Nivorn M., Kunawudhi A., Promteangtrong C., Boonkawin N., Jantarato A. (2020). Evaluation of Imaging Windows for Tau PET Imaging Using ^18^F-PI2620 in Cognitively Normal Individuals, Mild Cognitive Impairment, and Alzheimer’s Disease Patients. *Molecular Imaging*.

[16] Iqbal K., Liu F., Gong C.-X., Grundke-Iqbal I. (2010). Tau in Alzheimer disease and related tauopathies. *Current Alzheimer Research*.

[17] Barret O., Seibyl J., Stephens A. (2017). Initial clinical pet studies with the novel tau agent 18-F Pi-2620 in Alzheimer’s disease and controls. *Journal of Nuclear Medicine*.

[18] Stephens A., Seibyl J., Mueller A. (2018). Ic-P-220: clinical update: ^18^f-Pi-2620, a next generation tau pet agent evaluated in subjects with Alzheimer’s disease and progressive supranuclear palsy. *Alzheimer’s & Dementia*.

[19] Villemagne V., Dore V., Mulligan R. (2018). Evaluation of 18F-PI-2620, a second-generation selective tau tracer for the assessment of Alzheimer’s and non-Alzheimer’s tauopathies. *Journal of Nuclear Medicine*.

[20] Mormino E. C., Toueg T. N., Azevedo C. (2020). Tau PET imaging with ^18^F-PI-2620 in aging and neurodegenerative diseases. *European Journal of Nuclear Medicine and Molecular Imaging*.

[21] Hsieh C.-J., Lin K.-J., Huang C.-C. (2013). Improved quantitation accuracy for 18F-florbetapir imaging by a subject-specific cortex mask. *Journal of Nuclear Medicine*.

[22] Joshi A., Navitsky M., Kennedy I., Mintun M., Pontecorvo M., Devous M. (2015). Evaluation of regional distribution of the PET tau tracer 18F-AV1451 (also known as 18F-T807) using SUVr analysis and voxel wise parametric mapping. *Journal of Nuclear Medicine*.

[23] Betthauser T. J., Koscik R. L., Jonaitis E. M. (2020). Amyloid and tau imaging biomarkers explain cognitive decline from late middle-age. *Brain*.

[24] Pereira J. B., Harrison T. M., La Joie R., Baker S. L., Jagust W. J. (2020). Spatial patterns of tau deposition are associated with amyloid, ApoE, sex, and cognitive decline in older adults. *European Journal of Nuclear Medicine and Molecular Imaging*.

[25] Okamura N., Harada R., Ishiki A., Kikuchi A., Nakamura T., Kudo Y. (2018). The development and validation of tau PET tracers: current status and future directions. *Clin Transl Imaging.*.

[26] Lemoine L., Leuzy A., Chiotis K., Rodriguez-Vieitez E., Nordberg A. (2018). Tau positron emission tomography imaging in tauopathies: the added hurdle of off-target binding. *Alzheimer's & Dementia: Diagnosis, Assessment & Disease Monitoring*.

[27] Whitwell J. L., Graff-Radford J., Tosakulwong N. (2018). Imaging correlations of tau, amyloid, metabolism, and atrophy in typical and atypical Alzheimer’s disease. *Alzheimer's & Dementia*.

[28] Schmidt M. E., Chiao P., Klein G. (2015). The influence of biological and technical factors on quantitative analysis of amyloid PET: points to consider and recommendations for controlling variability in longitudinal data. *Alzheimer's & Dementia*.

